# Advancements in Characterization Techniques for Microemulsions: From Molecular Insights to Macroscopic Phenomena

**DOI:** 10.3390/molecules29122901

**Published:** 2024-06-18

**Authors:** Longfei Li, Jiepeng Qu, Weidong Liu, Baoliang Peng, Sunan Cong, Haobo Yu, Biao Zhang, Yingying Li

**Affiliations:** 1 Research Institute of Petroleum Exploration and Development, Beijing 100083, China; li_dragonfly@163.com (L.L.); jpqu@mail.ustc.edu.cn (J.Q.); pengbl@petrochina.com.cn (B.P.); cns69@petrochina.com.cn (S.C.); zhangbiao22@mails.ucas.ac.cn (B.Z.); yingyingli@petrochina.com.cn (Y.L.); 2 College of New Energy and Materials, China University of Petroleum-Beijing, Beijing 102249, China; yhb@cup.edu.cn; 3 National Elite Institute of Engineering, China National Petroleum Corporation (CNPC), Beijing 102200, China; 4 School of Rare Earth, University of Science and Technology of China, Hefei 230026, China

**Keywords:** microemulsions, characterization techniques, formation mechanisms, microstructural, molecule

## Abstract

Microemulsions are thermodynamically stable, optically isotropic, transparent, or semi-transparent mixed solutions composed of two immiscible solvents stabilized by amphiphilic solutes. This comprehensive review explores state-of-the-art techniques for characterizing microemulsions, which are versatile solutions essential across various industries, such as pharmaceuticals, food, and petroleum. This article delves into spectroscopic methods, nuclear magnetic resonance, small-angle scattering, dynamic light scattering, conductometry, zeta potential analysis, cryo-electron microscopy, refractive index measurement, and differential scanning calorimetry, examining each technique’s strengths, limitations, and potential applications. Emphasizing the necessity of a multi-technique approach for a thorough understanding, it underscores the importance of integrating diverse analytical methods to unravel microemulsion structures from molecular to macroscopic scales. This synthesis provides a roadmap for researchers and practitioners, fostering advancements in microemulsion science and its wide-ranging industrial applications.

## 1. Introduction

It is generally acknowledged that a microemulsion constitutes a thermodynamically stable solution system comprising oil, water, and amphiphilic solutes, exhibiting optical isotropy [1,2] and strong solubilization capabilities [3,4,5,6,7]. These versatile solutions are valuable across various industries, including pharmaceuticals, food, petroleum, and chemicals. With droplet sizes typically ranging from 10 to 100 nm [8,9], microemulsions possess the unique ability to achieve ultra-low interfacial tension, a property often leveraged to enhance processes such as oil recovery [10,11,12,13]. According to the IUPAC, the dispersed domain of microemulsions varies in diameter from approximately 1 to 100 nm, with a common range falling between 10 and 50 nm [14].

Microemulsions are conventionally categorized into four types: Winsor I (water-in-oil), Winsor II (oil-in-water), Winsor III (bicontinuous three-phase), and Winsor IV (bicontinuous single-phase) [15,16], each exhibiting significant differences in performance [11,17,18]. However, the complexity of these systems, influenced by numerous factors, poses challenges in maintaining stability and performance consistency. Further research is crucial to elucidating the phase transition processes, composition conditions, and properties of microemulsions, particularly in industries such as petroleum and pharmaceuticals, where precise control over structure is imperative. Understanding the structure of microemulsions is vital for optimizing processes such as drug delivery, formulation development [19], and oil recovery [20].

This review delves into the formation mechanisms, macroscopic types, microstructural characteristics, and characterization techniques of microemulsions. Additionally, it integrates multiple characterization methods and matches them to their applicable scale ranges, emphasizing the necessity of different analytical methods to reveal the structure of microemulsions from molecular to macroscopic scales. This comprehensive approach aims to promote a multidimensional understanding of the structural characteristics of microemulsions and provide a step-by-step roadmap for researchers. Through this exploration, this article aims to contribute to the advancement of microemulsion research and its applications in various industries.

## 2. The Formation Mechanism of Microemulsions

### 2.1. The Instantaneous Negative Interfacial Tension Theory

The transient negative interfacial tension theory suggests that while surfactants can greatly reduce the oil–water interfacial tension, the decrease is not sufficient to form microemulsions [21,22]. The addition of an appropriate co-surfactant, which mixes and adsorbs with the surfactant, further decreases the oil–water interfacial tension. The monolayer formed by the surfactant at the oil–water interface can create a membrane pressure that reduces the interfacial tension. Moreover, when co-surfactants are present, their distribution in the oil and water phases leads to a substantial reduction. This can decrease the system’s interfacial tension to 10^−3^–10^−5^ mN/m, or even to negative values, resulting in transient negative interfacial tension.

(1) $$-\mathrm{dy}=\Sigma \Gamma_{i}d\mu_{i}=\Sigma \Gamma_{i}\mathrm{RT}\mathrm{dln}c_{i}$$

(2) $$\gamma =\gamma_{O/W}-\pi$$

where γ represents the oil–water interfacial tension, Γ_i_ represents the adsorption quantity of component i, μ_i_ represents the chemical potential of component i, and c_i_ represents the bulk-phase concentration.

However, negative interfacial tension does not naturally exist; this would lead to the system spontaneously expanding, causing the interfacial tension to rise to equilibrium, stabilizing at a minimal positive value. Surfactants and co-surfactants adsorb at the oil–water interface, creating a microemulsion system. This theory offers a partial explanation of the microemulsion formation mechanism, but negative interfacial tension cannot be experimentally measured and thus cannot be verified. Additionally, this theory fails to account for the formation of different types of microemulsions.

### 2.2. The Double-Layer Membrane Theory System

The double-layer membrane theory [23] proposes that a mixed membrane formed by the synergistic action of surfactants and co-surfactants adsorbs at the oil–water interface, creating a highly flexible double-layer membrane (also known as the third phase or intermediate phase). This membrane spontaneously bends at the oil–water interface due to varying concentration ratios of system components, with the direction of bending determining the type of microemulsion. The double-layer membrane theory does not account for factors such as temperature, pressure, and composition and lacks explanations for dynamic processes and molecular-level interactions. As a result, theories such as the geometric arrangement theory [24] and the R-ratio theory [25] have been developed to explain microemulsion formation from spatial and molecular interaction perspectives, respectively.

The former theory posits that the polar head of the surfactant and the alkyl chain form two uniform interfaces with water and oil. It also considers the geometric packing of surfactant molecules on the interface membrane, introducing the filling coefficient P:

(3) $$P=\frac{V}{a_{o}l_{c}}$$

The former theory introduces the concept of the filling coefficient P, where V represents the volume of the alkyl chain in surfactant molecules, a_o_ represents the optimal cross-sectional area of each surfactant polar head on the plane, and l_c_ represents the length of the alkyl chain. When P = 1, the interface is not curved, forming a lamellar liquid crystal phase. When P > 1, the cross-sectional area of the alkyl chain is maximized, and the interface membrane points toward the oil phase, forming a W/O-type microemulsion. Conversely, when 1/3 < P < 1, an O/W-type microemulsion is formed, and when P < 1/3, only micelles are formed rather than microemulsions. The geometric arrangement theory further refines the double-layer membrane theory by introducing molecular-scale parameters.

The latter theory relates the structure and properties of microemulsions to the ratio of cohesive energies, defined as R:

(4) $$R=\frac{A_{\mathrm{SO}}-A_{\mathrm{OO}}-A_{\mathrm{ii}}}{A_{\mathrm{SW}}-A_{\mathrm{WW}}-A_{\mathrm{hh}}}$$

**Figure 1:**
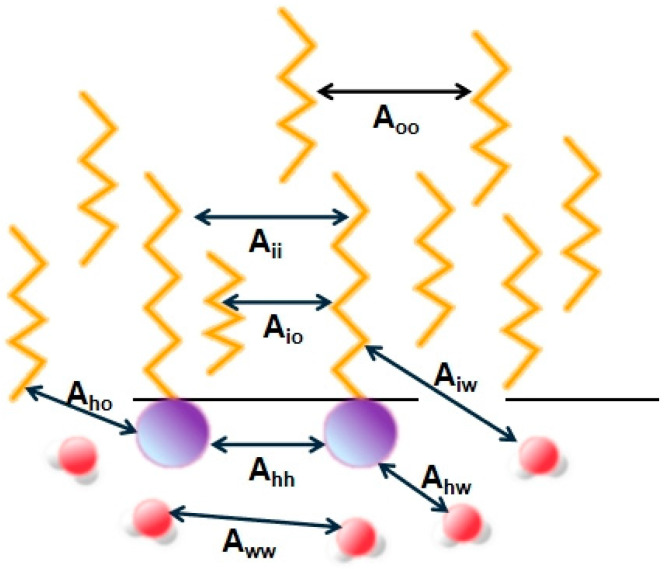
The diverse molecular interaction energies across different molecular types [26].

The formula describes A_SO_ as the cohesive energy between the hydrophobic group of the surfactant and oil molecules, A_OO_ as the cohesive energy between oil molecules, A_ii_ as the cohesive energy between hydrophobic groups, A_SW_ as the interaction energy between hydrophilic groups and water, A_WW_ as the interaction energy between water molecules, and Ahh as the cohesive energy between hydrophilic groups, as shown in Figure 1.

Combining the double-layer membrane theory, when R = 1, the interface does not curve, and the system exhibits a bicontinuous structure. When R < 1, the interaction force between the interface and the oil phase is stronger, causing the interface to bend toward the water phase to achieve equilibrium, forming an O/W-type microemulsion. Conversely, when R > 1, the interaction force between the interface and the water phase is stronger, causing the interface to bend toward the oil phase and making it easier to form a W/O-type microemulsion.

### 2.3. The Micellar Solubilization Theory

**Figure 2:**
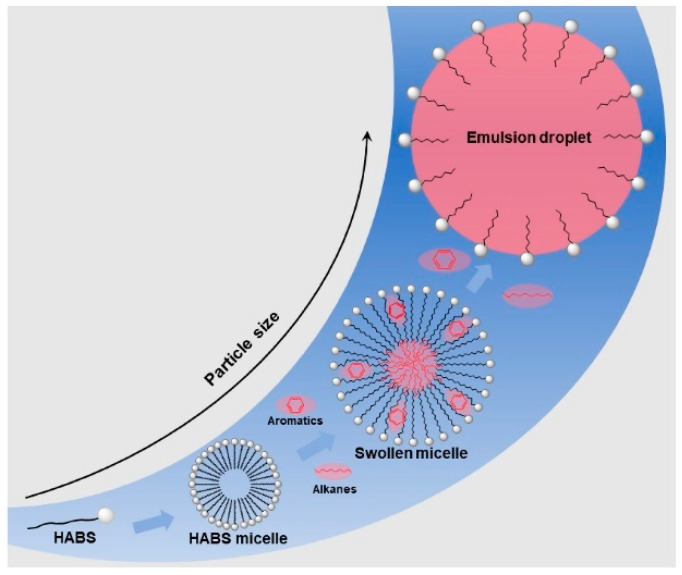
Solubilization behavior and droplet size change of surfactant aggregates [28].

The micellar solubilization theory [27] posits that microemulsions are swollen micellar systems resulting from micellar solubilization. Their droplet size falls between emulsions and micelles, with micelles solubilizing oil–water to form microemulsions, referred to as “solubilized micelles”. When the concentration of surfactants in the solution exceeds the critical micelle concentration (CMC), surfactants spontaneously form micelles. Micelles exhibit strong solubilization capabilities, leading to the formation of microemulsions after solubilizing a certain amount of oil or water. The concept illustrated in Figure 2 is consistent with the aforementioned idea. This theory provides a more intuitive description of the microemulsion formation process but is limited to microemulsion systems where surfactants are amphiphilic substances, and it lacks the precise determination of microemulsion size.

Each of the above theories, to some extent, explains the formation of microemulsions. However, the first two theories lack robust empirical evidence in explaining auto-emulsification phenomena, whereas the micellar solubilization theory has broader applicability and can vividly depict the transition in microemulsion microstructures.

### 2.4. Electrochemical Theory and System Stability

Understanding the role of electrochemical phenomena in microemulsion systems is essential when discussing the formation mechanism of microemulsions. Consequently, the Debye–Huckel, Gouy–Chapman, and Stern layer theories offer a comprehensive insight into these phenomena by applying various approximation methods to solve the Poisson–Boltzmann equation. The Debye–Huckel theory is applicable to dilute solutions, describing the electrostatic interactions between ions in solution by simplifying the Poisson–Boltzmann equation [29]. This theory provides an estimation of the relationship between ionic strength and electric potential in microemulsions, aiding in explaining the impact of charge shielding and the ionic atmosphere on microemulsion formation. The Gouy–Chapman theory broadens the understanding of the electric double layer, detailing the distribution of ions in the diffuse layer [30]. By solving the Poisson–Boltzmann equation numerically, the Gouy–Chapman theory offers models for the distribution of potential and charge density. This analysis is crucial for understanding the charge distribution and potential gradient at the microemulsion interface and the adsorption behavior of surfactant molecules [31]. However, the Gouy–Chapman model presumes an infinitely large flat charged surface and considers ions in solution as point charges, assumptions not entirely valid in actual reverse micelle systems. The Stern layer model revises the Gouy–Chapman theory, taking into account the potential changes caused by the size of ions fixed on the surface and their specific adsorption effects at the interface. This model splits the electric double layer into a compact layer adjacent to the interface and a diffuse layer outside, detailing the electrochemical properties at the interface [32]. This detailed model can predict the electric double-layer capacitance and potential distribution in microemulsion systems more precisely, elucidating how interface charges and surfactant molecule interactions affect the stability and formation process of microemulsions. These theories elucidate the electrochemical phenomena involved in the formation mechanism of microemulsions by solving the Poisson–Boltzmann equation, offering theoretical backing for understanding the structure and stability of microemulsions.

## 3. The Types of Microemulsions and Their Microstructures

**Figure 3:**
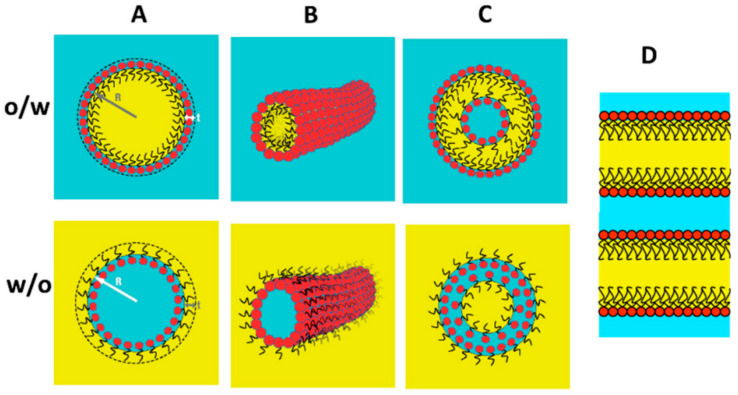
Schematic diagram of microemulsion self-assembled surfactant structure: (**A**) spherical micelles; (**B**) cylindrical micelles; (**C**) vesicles; (**D**) bicontinuous planar layers [35].

Microemulsions of the surfactant type bear structural similarities to traditional surfactant micelles. In conventional surfactants, there are spherical, cylindrical, and rod-shaped micelles [33,34]. By introducing oil into the polar region, oil-in-water (O/W)-type microemulsions are formed, while introducing water into the non-polar region leads to water-in-oil (W/O) microemulsions. The former, when only a small amount of oil is dissolved, is referred to as water-in-oil-type micelles or direct micelles. Conversely, the latter, when only a small amount of oil is dissolved, is termed oil-in-water-type micelles or reverse micelles. Figure 3 depicts a schematic diagram of the aforementioned process.

In water, surfactants can also form structures such as vesicles, liquid crystal bilayers, or other cubic bicontinuous phases based on planar or locally planar double-layer structures [36]. Additionally, when the hydrophobic tails of surfactants encounter oil, the bilayer transitions into two single layers separated by an oil layer, significantly reducing the rigidity of the entire molecular layer. The entropy of locally planar surfactant membranes gradually transforms the liquid crystal phase regions into isotropic liquid structures, where the water phase and oil phase intertwine through surfactant monolayers, collectively known as bicontinuous microemulsions [35]. Since bicontinuous structures exist in three-dimensional space, their structure is often understood and studied through molecular computational simulations.

Compared to O/W- or W/O-type microemulsions, bicontinuous phase microemulsions exhibit ultra-low interfacial tension between oil and water, resulting in a significantly increased interface area, allowing for the substantial solubilization of oil and water. Their microstructure is highly complex [37]. This characteristic finds widespread application in enhancing oil recovery rates.

## 4. Microemulsion Characterization Techniques

Certainly, detailing the characterization techniques for microemulsion structures is crucial for understanding their formation mechanisms, future development, and practical applications.

### 4.1. Chemical Composition—Component Analysis

**Figure 4:**
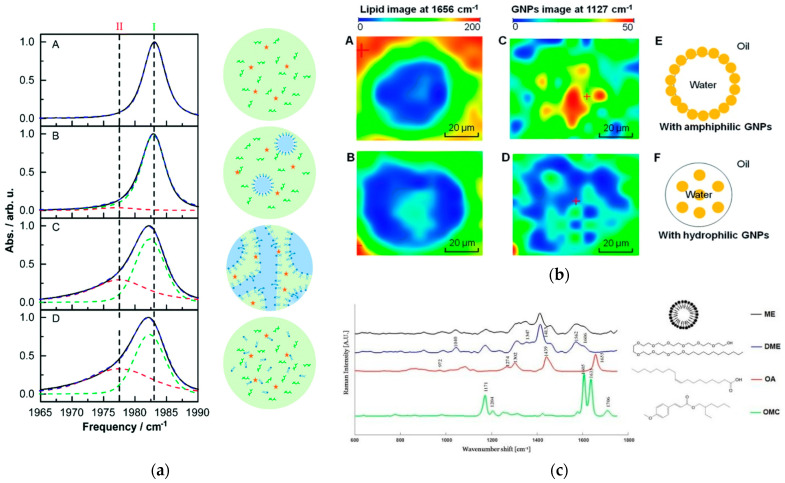
(**a**) FTIR spectra showing the stretching mode of C≡O in different liquid phases [42]. (**b**) Raman imaging of different substances (**A**–**D**) and the formation diagram of microemulsions (**E**,**F**). (**c**) Raman spectra of different substances and microemulsion formation [43,44].

Spectroscopic techniques are based on the interaction between matter and electromagnetic waves. They are used to analyze and identify the structure and composition of substances by measuring their absorption, scattering, or emission behavior across different wavelengths of electromagnetic waves. The main techniques include Fourier-transform infrared spectroscopy (FTIR), Raman spectroscopy (RS), and energy-dispersive X-ray spectroscopy (EDS), among others. These techniques utilize the distinct characteristics of electromagnetic waves from infrared to X-ray regions to detect electronic transitions, changes in vibrational modes, and differences in the characteristic energy levels of elements, thus studying the microscopic properties and macroscopic manifestations of substances. FTIR can investigate the molecular structures and the state of dissolved water in microemulsions, elucidating the intermolecular interactions, solvation effects, and component distributions within these systems [38]. Due to the broad water background signal, infrared spectroscopy of microemulsions, which are typically formed with water and oil, can be difficult. Conversely, water exhibits a very low Raman signal, often negligible at low wavenumbers, whereas the unsaturated C=C bonds in oil show a strong Raman response, making RS appropriate for microemulsion studies [39]. The Raman signal intensity of the internal water phase was measured under various refractive index combinations, summarizing the effect of liquid phase refractive index matching and facilitating the study of W/O/W-type droplets [40]. EDS spectral analysis provides qualitative and quantitative analysis of elements in microemulsion components, deepening understanding of their chemical composition and structure. This technique analyzes the presence of elements such as sulfur and oxygen in microemulsions and is combined with scanning electron microscopy to analyze the location and role of surfactants in microemulsions [41]. Figure 4 illustrates the application of FTIR and Raman spectroscopy in the study of microemulsions.

Fluorescence Correlation Spectroscopy (FCS), a highly sensitive optical method, shows considerable benefits in the analysis of molecular dynamics and concentration variations [45]. FCS, compared to other spectroscopic methods, offers extremely high sensitivity, enabling the detection of single-molecule fluorescence signals, which is ideal for low-concentration-sample studies. Additionally, FCS can track dynamic behaviors like molecular diffusion, interactions, and reaction processes in real time, delivering dynamic data unattainable by other static spectroscopic techniques. Furthermore, FCS provides high spatial resolution, enabling measurements in very small observation volumes, which is ideal for the local analysis of complex microenvironments [46]. By choosing various fluorescent probes, FCS can be broadly used to investigate the dynamic behavior and interactions of amphiphilic molecules like surfactants. In summary, FCS’s advantages in high sensitivity, real-time dynamic analysis, non-destructive measurements, small sample volumes, high spatial resolutions, and versatility make it a powerful tool for researching molecular dynamics and interactions, particularly in low-concentration and complex environments [47].

While spectroscopic analysis techniques have strong capabilities in terms of microemulsion characterization, they are limited by sensitivity, resolution, complex sample preparation, and data analysis challenges. Future efforts can focus on improving equipment performance, simplifying sample preparation and measurement processes, and utilizing artificial intelligence to optimize data processing. The aim of such efforts is to make spectroscopic analysis methods more accurate, user-friendly, and widespread, supporting in-depth research and applications of complex microemulsion systems.

### 4.2. Intermolecular Interaction and Aggregation

#### 4.2.1. Nuclear Magnetic Resonance (NMR)

**Figure 5:**
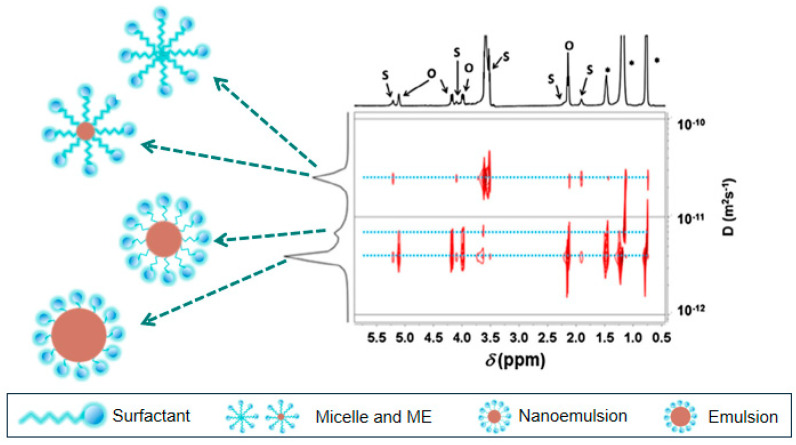
NMR for characterizing the structure of a microemulsion [69].

NMR is a non-invasive research method that can be used to analyze the structure of substances and provide kinetic information about molecular interactions, with its applications most often seen in proton and carbon spectra. This technology is widely used to determine the structure of microemulsions [48], study the effects of different components on microemulsions, and assist in constructing phase diagrams, as shown in Figure 5 [49,50]. Here, we explain diffusion nuclear magnetic resonance (dNMR) based on pulsed magnetic gradient techniques, especially pulsed field gradient NMR (PFG NMR) [51], pulsed-gradient spin-echo NMR (PGSE NMR) [52], and diffusion-ordered spectroscopy (DOSY) [53,54]. In cases where unexpected diffusion occurs in NMR samples, spatially encoded diffusion NMR (SPEN DNMR) techniques have also been developed [55].

dNMR technology determines the self-diffusion coefficients of different chemical substances by identifying their nuclear magnetic resonance signals, providing insights into the composition distribution and microstructure of complex solutions, and further studying the dynamics of microemulsion systems [56,57]. Depending on the type of microemulsion structure, different self-diffusion coefficients are observed; for example, in O/W-type microemulsions, the self-diffusion coefficient of water is much greater than those of surfactants and oil. In W/O-type microemulsions, the self-diffusion coefficient of oil is much greater than those of surfactants and water. For double continuous structures, the self-diffusion coefficient of surfactants is much lower than those of oil and water, and the self-diffusion coefficients of oil and water are close to those of pure components (or may experience a sharp loss of nuclear magnetic resonance signal, depending on the internal structure and relaxation time of the double continuous phase) [58,59].

PFG NMR introduces pulse gradients during the acquisition of nuclear magnetic resonance signals, creating phase differences among nuclear magnetic signals from different locations. Based on the different echo attenuation characteristics of each nuclear magnetic resonance signal, it monitors the self-diffusion coefficients of different components in the same system and tracks the self-diffusion paths of molecules. PFG NMR can determine the relationship between the molecular transport rate and distance in microemulsion systems. By measuring the average displacement of molecules, the self-diffusion coefficients of different components can be obtained [60].

PGSE NMR is based on PFG and introduces spin echoes during the acquisition of nuclear magnetic resonance signals to measure molecular self-diffusion behavior [61]. This labeling does not affect the microscopic dynamics of molecules but provides information about the time-dependent probability distribution of molecular displacements. By measuring the decay of nuclear magnetic resonance signals, the self-diffusion coefficients of different components can be obtained. PGSE NMR can be used to analyze component distributions and microstructures in microemulsion systems [62]. However, the sample must be in a liquid state, and the requirements for the instrument are higher.

DOSY is one of the methods proposed by Morris and Johnson for processing and representing information recorded in NMR experiments [63]. It outputs two-dimensional spectra, with one dimension related to chemical shifts and the other representing diffusion coefficients. DOSY can not only identify different components of microemulsions but also provide information about system dynamics (restrictions, chemical exchange rates, etc.) due to the time dependence of diffusion coefficients [64]. Furthermore, DOSY NMR technology has the advantages of low sensitivity to light scattering, low sample requirements, and the ability to detect characteristic spectra of turbid samples [65].

In summary, nuclear magnetic resonance technology has made significant progress in studying the microstructures of microemulsion systems. It can not only determine the diffusion behavior of microemulsion droplets in complex emulsion systems but also be used to analyze the component distribution and microstructure based on different self-diffusion coefficients and chemical shifts [66], aiding in the study of dynamic information about internal surfactants [67,68]. In practical applications, there is still a need to establish clear relationships between microemulsion systems and the pulse sequences used, often requiring multiple attempts at matching.

#### 4.2.2. Small-Angle Scattering

Small-angle scattering techniques include small-angle X-ray scattering (SAXS) and small-angle neutron scattering (SANS), both of which have been widely applied to investigate the microstructure of microemulsions. By analyzing their scattering profiles, information such as morphology, size, and even phase transitions can be obtained [70], leading to insights into the size and electron density of hydrocarbon chains and hydrophilic groups [71,72,73].

**Figure 6:**
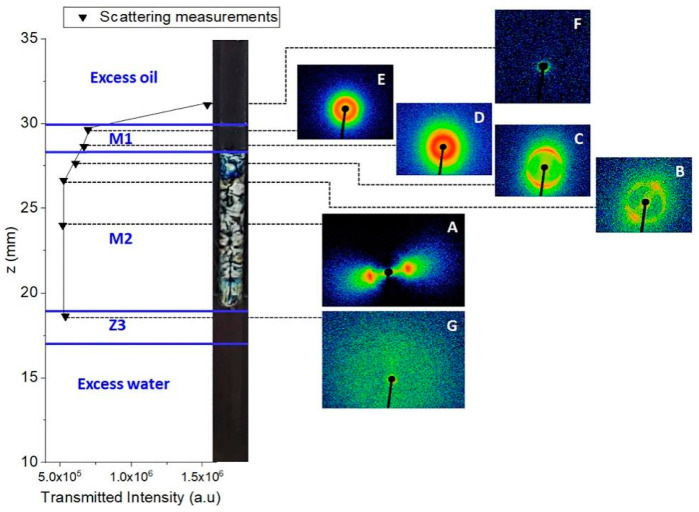
The different phases of microemulsion systems can be visually represented using a SAXS schematic diagram, The images labeled (**A**–**G**) on the right side are scattering patterns obtained at different positions along the column [68].

SAXS relies on fluctuations in electron density within the range of 1 to 100 nm in microemulsion systems. The scattering intensity is influenced by factors such as droplet size, shape, dispersion, orientation, and electron density distribution, resulting in different scattering functions [74]. Utilizing SAXS to characterize microemulsion systems involves obtaining scattering intensity and scattering vector maps. By combining sphere shell models and cylinder shell models for data fitting, the impact of surfactant structures on the diffusion layer properties and micelle bodies of microemulsions can be analyzed [75]. Moreover, SAXS can also be used to determine the type of microemulsion, as shown in Figure 6, especially the double continuous phase structure and existing structural gradients [68], which is crucial for studying phase transitions in microemulsion systems.

**Figure 7:**
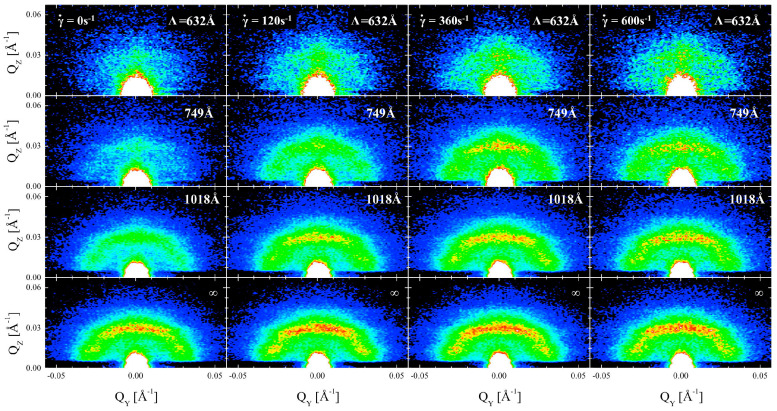
Grazing-incidence small-angle neutron scattering (GISANS) diagram of microemulsions [79].

Compared to SAXS, SANS is sensitive to light elements, isotopic labeling, and magnetic moments, allowing it to measure nano- to micrometer-scale microstructures. By selecting appropriate isotopes to match, more interface details can be explored. When analyzing O/W or W/O microemulsion systems using SANS, the scattering profile is typically decomposed into the product of two angular functions—intra-droplet interference and inter-droplet interference—for analytical fitting. Alternatively, indirect Fourier transforms of scattering data can be used to obtain distance distribution functions for geometric calculations [76]. Various model approaches can yield information such as the interaction radius [58], droplet volume fraction [77], and polydispersity [78]. Figure 7 demonstrates the application of SANS in the study of microemulsions.

Combining SAXS and SANS with other techniques allows for fitting the analysis of the complex internal structure of bicontinuous microemulsions using mathematical models [80,81]. In practical applications, the accuracy and applicability of data models in small-angle scattering are crucial [82,83,84]. Utilizing algorithms based on machine learning and artificial intelligence for mathematical models in microemulsion systems will be a precise and effective direction.

#### 4.2.3. Dynamic Light Scattering (DLS)

DLS, also known as Photon Correlation Spectroscopy (PCS) or Quasi-Elastic Light Scattering (QELS), is an optical diffusion measurement technique used to study the average droplet size and size distribution of droplets in fluids. It is based on the Doppler frequency shift of scattered light caused by the Brownian motion of droplets in the liquid, resulting in fluctuations in the intensity of scattered light. By analyzing the decay rate of the autocorrelation function (ACF) of intensity, the diffusion coefficient Dm between droplets and solvent molecules or collective diffusion information can be obtained, providing dynamic information about the droplets [85].

**Figure 8:**
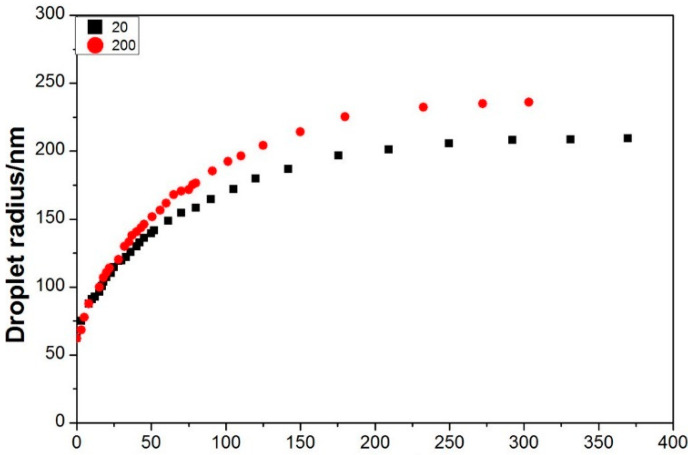
The relationship between microemulsion droplet size and time [97].

For microemulsion systems, DLS primarily measures the droplet size information of micelles within the microemulsion system, including the hydrodynamic diameter [86], Particle Dispersion Index (PDI) [87], relaxation time [68], etc. It is also used to investigate behaviors such as droplet diffusion and coalescence under different conditions [77], analyzing the impact of different times or temperatures on system stability. When studying the dispersed phase in a continuous phase, i.e., O/W- or W/O-type microemulsion systems, DLS can effectively determine the system’s composition under simple conditions. In W/O-type microemulsions, where the oil is the polar continuous phase, droplets can be approximated as hard spheres, similar to non-ionic surfactant O/W types, allowing for the determination of micelle size through self-diffusion and mutual diffusion combined with dNMR [88,89,90]. However, for O/W-type microemulsions prepared with ionic surfactants, the situation is more complex due to mutual repulsion effects and cannot be approximated using a hard-sphere model. Instead, combining dNMR and DLS techniques can provide information about the interaction and micelle size based on self-diffusion and mutual diffusion coefficients in O/W-type microemulsions [91,92]. Additionally, DLS can measure the average intensity of scattered light, allowing for the assessment of interactions between droplets in microemulsion systems [89,90]. Figure 8 illustrates the application of DLS in characterizing the size of microemulsions.

DLS is an effective technique for characterizing size information in microemulsion systems. However, it is challenging to assess double continuous phase structures. It can only characterize physical sizes and is influenced significantly by dust droplet scattering [93], high sensitivity to larger droplets [94,95], or optical matching points [96]. Moreover, excessive dilution during experimentation may alter the properties of microemulsion systems or even cause phase transitions, leading to significant errors in information retrieval. Simplifying the DLS sampling process and adopting new sampling methods or detection approaches to prevent the influence of excessive dilution, excessive shear forces, or electrolytes on microemulsion systems is necessary.

#### 4.2.4. Cryo-Electron Microscopy (Cryo-EM)

An electron microscope (EM) employs a high-speed electron beam instead of traditional visible light to illuminate the target sample, analyzing the signals produced by the interaction of electrons with the sample to construct images. This technology is mainly divided into two categories: transmission electron microscopes (TEMs) and scanning electron microscopes (SEMs). TEMs use an electron beam to penetrate thin samples, capturing transmitted electrons to obtain high-resolution images of the sample’s interior; in contrast, SEMs scan the electron beam across the sample surface, detecting secondary electrons or backscattered electrons to generate three-dimensional images of the sample surface.

Since microemulsions are liquids, they cannot be directly observed using SEMs or TEMs. During observation, the sample must be solidified before being placed in a vacuum pump. Depending on the solidification method, electron microscopy techniques can be further classified into Cryogenic Transmission Electron Microscopy (Cryo-TEM), Cryogenic Scanning Electron Microscopy (Cryo-SEM), and freeze-etching (also known as freeze-fracture) or freeze-replica microscopy. This technology is the only one that can directly observe the microscopic morphology of microemulsions [98,99,100].

**Figure 9:**
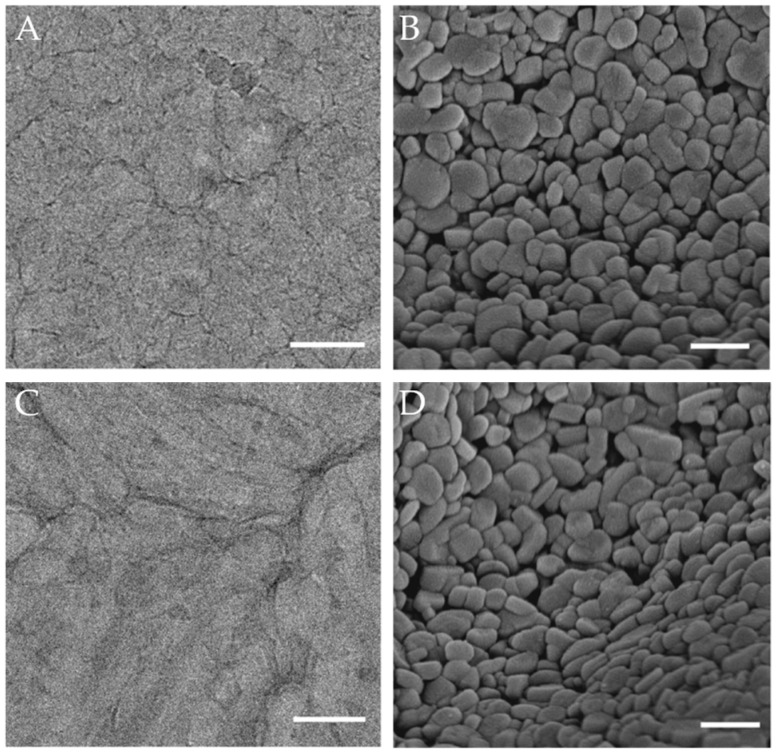
Microstructure of microemulsion under freezing transmission electron microscope (**A**,**C**) and freezing scanning electron microscope (**B**,**D**) [107].

In the study of microemulsions, cryo-electron microscopy can provide high-resolution images of microemulsion droplets, allowing researchers to visually analyze the size, shape, and surface characteristics of droplets, identify interfaces and structural organization between different phases, and study phase transitions and dynamic behaviors of microemulsions under changing conditions [101,102]. Figure 9 shows the microstructure of a microemulsion observed under cryo-electron microscopy. Cryo-SEM can reveal the size of nanoemulsion structures on frozen fracture surfaces and is correlated with DLS, revealing that the addition of surfactants can widen the size and structural distribution [103]. Cryo-TEM can observe the droplet structure in surfactant-free microemulsions, confirming the existence of bicontinuous phase microemulsions [104]. To address the issue of electron microscopy providing only static information about samples, liquid-phase transmission electron microscopy (LPTEM) has been developed and successfully used to observe changes in microemulsion droplets due to formula changes in undisturbed conditions [105]. Nonetheless, steps like thin-film preparation and rapid freezing during sample preparation may alter the original state of the microemulsion. Moreover, when the sample has a complex bicontinuous structure, resolution limitations may prevent the accurate capture of the sample’s true structural state.

To address these challenges, researchers can employ gentler sample preparation techniques, such as improved rapid freezing methods, to more effectively preserve the initial state of microemulsions and avoid the formation of ice crystals, thus reducing interference and damage to samples. It is worth noting that Environmental Scanning Electron Microscopy (ESEM) technology enables the observation of samples in nearly natural states, including in wet or specific gas environments [106]. By further refining these techniques, more realistic observation conditions can be provided for liquid samples like microemulsions. Additionally, combining these techniques with other characterization methods for comprehensive quantitative analysis can further deepen researchers’ understanding of microemulsion systems.

### 4.3. Phase Characteristics

#### 4.3.1. Conductometry

**Figure 10:**
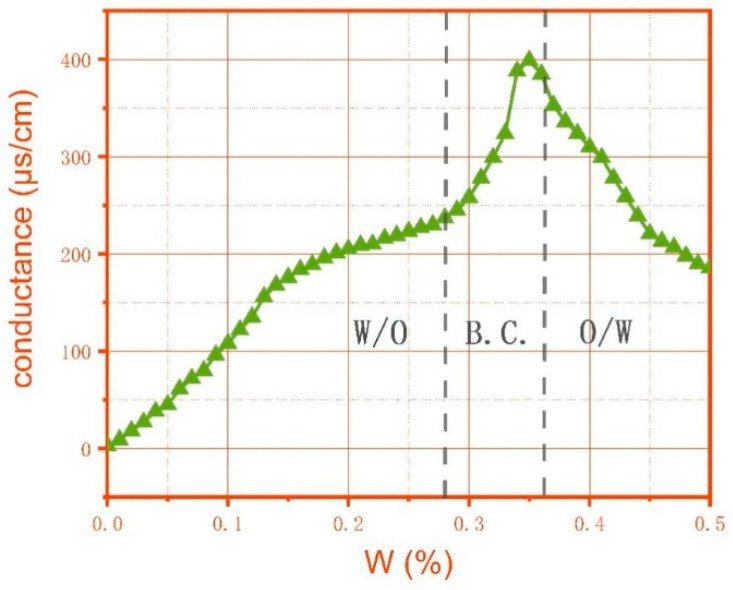
Relationship between water phase and electrical conductivity in microemulsion [111].

Conductometry not only serves as a direct means to discern the type of microemulsion but also stands as an incredibly convenient testing approach. When the surfactant in the microemulsion system is an ionic surfactant or contains salts, changes in the conductivity gradient can be used to distinguish between different structures. Typically, the system’s conductivity increases with rising water content, sharply rising as the water concentration reaches the percolation threshold [108] and then leveling off, forming an S-shaped curve. Consequently, the conductivity of W/O-type microemulsions is the lowest among all microemulsion structures, increasing as the water content rises. When the water content reaches a certain level, the interaction between water droplets within the microemulsion system, facilitated by charge hopping or the instantaneous aggregation of connected droplets with water nuclei, enhances the interaction between water domains. This may lead to the formation of a continuous water phase in the oil phase, known as a bicontinuous phase, which exhibits conductive connectivity, thereby accelerating the rate of the conductivity increase. The magnitude of this increase is related to the internal water (or saltwater) content and distribution domain of the structure [109]. The application of conductivity based on the aforementioned principles is shown in Figure 10. Additionally, some scholars have developed differential electric analyses based on the conductivity method to determine the diffusion coefficient of microemulsion droplets and detect the microstructural transformation in the microemulsion region [110].

While the conductivity method is convenient and can identify microemulsion types, it falls short in determining microstructural aspects such as micelle types, sizes, and bicontinuous phase structures. Moreover, in microemulsion systems where surfactants and hydrocarbons are present, the solution’s internal resistance can be high, requiring sufficient electrolytes to meet measurement standards. These electrolytes can alter the system’s properties, leading to significant measurement biases. Combining analytical methods to process conductivity data and obtain more precise structural insights represents a breakthrough direction for the conductivity method in measuring microemulsions.

#### 4.3.2. Zeta Potential

The zeta potential, also known as the electrokinetic potential, is measured based on the electrophoresis effect, which relates the velocity of charged droplets under an electric field to the zeta potential. Major measurement techniques include electrophoretic light scattering (ELS), micro-electrophoresis, and phase analysis light scattering (PALS). During the formation of microemulsions, changes in factors such as surfactants, system salinity, and temperature can lead to the formation and growth of micelles. Therefore, the stability of micelle structures directly determines the performance of microemulsion systems. By measuring the zeta potential, one can predict the long-term stability of microemulsions, adjust formulations to optimize stability and functionality, evaluate the effectiveness of surfactants, gain a deeper understanding of droplet interactions, and adapt to environmental factors affecting the stability of microemulsion systems, all of which are crucial for ensuring the performance of microemulsions [112,113].

Nevertheless, in practical applications, microemulsion systems are commonly employed at high salt concentrations. Since electrolytes can screen the surface charges of droplets and modify electrophoretic behavior, the concentrations and types of electrolytes in the solution have a substantial impact on zeta potential measurements.

#### 4.3.3. Refractive Index (RI)

RI measurement is a physical technique for measuring the effect of a substance on the propagation speed of light waves associated with the medium’s electronic structure and molecular arrangement. In microemulsion systems, refractometry assists in identifying the current phase state of the system, with distinct phase states corresponding to different refractive index values related to the microemulsion’s composition and dispersed-phase properties. Near the critical point, the refractive index of the microemulsion system can undergo significant changes, potentially linked to variations in the system’s density or electric field. By measuring refractive index variations with parameters such as temperature and pressure, the critical point of the system, i.e., the temperature or pressure at which phase separation occurs, can be identified. These variations are closely tied to the microemulsion’s microstructure and the behavior of the surfactants [114].

Utilizing specific techniques to determine the microemulsion’s critical concentration and critical temperature, the coexistence curve of temperature and the refractive index can be converted to the coexistence curve of temperature and the volume fraction (T, φ), enabling a quantitative analysis of the microemulsion system’s critical behavior, including critical temperature, critical exponent, and critical amplitude. This facilitates the comprehension of the microemulsion system’s thermodynamic properties, such as droplet interactions and system stability [115]. Refractometry is a non-destructive testing method that does not harm the sample, is straightforward and rapid to perform, and can assess the stability of a microemulsion system. However, this characterization method necessitates that the sample be uniform and bubble-free, environmental conditions remain stable, and the measurement technique is compatible with the sample’s optical properties. Improving sample preparation and refractometry techniques to maintain stability throughout the measurement process is vital when adapting testing methods to various optical properties of systems, which enhances their applicability.

#### 4.3.4. Differential Scanning Calorimetry (DSC)

DSC is a thermal analysis technique that measures the energy difference between a sample and a reference as the temperature changes under programmed heating conditions [116]. The curve recorded by the differential scanning calorimeter is known as the DSC curve, with the sample’s heat absorption or release rate, i.e., the heat flow rate dH/dt (in millijoules per second), on the vertical axis and temperature T or time t on the horizontal axis, enabling the measurement of multiple thermodynamic and kinetic parameters.

This technique is extensively applied to examine the low-temperature behavior of surfactant-based microemulsion systems [117]. This is due to the varying melting points (freezing points) of different types of water in microemulsions: free water melts at 0 °C; interfacial water, confined within the dispersed system, melts around −10 °C; and bound water, associated with hydrophilic groups, melts below −10 °C. Water undetectable by DSC is termed “non-freezable” water. By identifying the freezing peaks of various water types in the microemulsion system, the phase types can be determined, and the system’s thermal behavior at low temperatures can be monitored, distinctly differentiating the interactions between water and surfactants, co-surfactants, and other components at the interface and within the system, thus elucidating the transitions in the system’s crystalline structure. This approach substantially aids in comprehending the behavior of the aqueous phase in systems with restricted fluidity and strong interactions.

However, this method necessitates the microemulsion system to be at low temperatures, where temperature fluctuations can induce structural and phase changes in the system, permitting the study of the system’s state in dynamic equilibrium within a specific temperature range. Additionally, research on this system predominantly focuses on non-ionic surfactant microemulsion systems, given that interactions in ionic surfactant microemulsion systems are more complex, presenting challenges for the extended application of DSC. Podlogar et al. [118] developed an artificial neural network (ANN) coupled with genetic algorithms to manage complex intermolecular interactions, directly identifying the type of microemulsion from DSC curves. The ongoing advancement of this computational model shows potential for extending the interpretation of DSC for microemulsion systems to higher temperature ranges.

**Figure 11:**
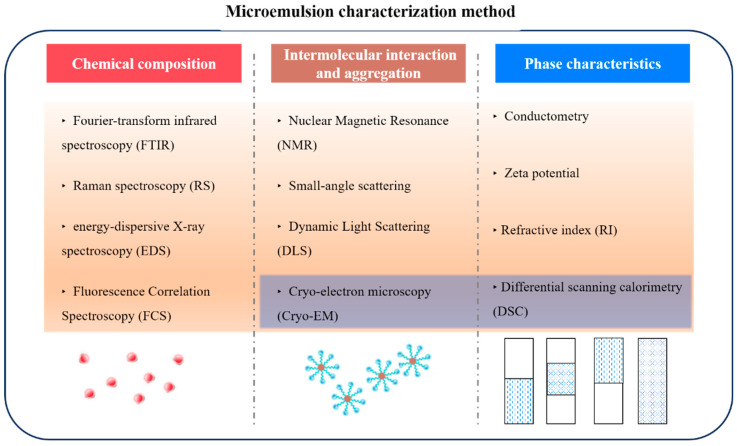
Microemulsion multi-scale characterization technology roadmap (the blue-background techniques are suitable for low-temperature conditions).

Microemulsions are thermodynamically stable systems; however, their internal composition is complex and sensitive to external factors. Thus, characterizing them using multiple techniques at different scales (including molecular, micelle, and macroscopic phases) can provide complementary chemical, size, thermodynamic, and kinetic information, compensating for each technique’s limitations at other scales. As shown in Figure 11, this study presents a roadmap for standard microemulsions based on different scales, considering accuracy, applicability, and portability. It is recommended to select 2–3 characterization techniques for each scale, from top to bottom, to ensure the stability and accuracy of the results. Notably, Cryo-EM and DSC have stringent temperature requirements.

## 5. Conclusions and Prospects

### 5.1. Conclusions

In the field of microemulsion research, the comprehensive use of various characterization techniques is key to achieving a full understanding of microemulsion properties from the molecular and atomic scale to the aggregate scale. Spectroscopic analysis can provide chemical information about the composition of microemulsions, including specific functional groups and molecular dynamics of surfactants and the oil phase, although it is limited in its ability to analyze complex systems, and infrared signals are easily affected by the strong absorption of water. NMR technology is regarded as a potent tool due to its ability to provide detailed information on molecular dynamics, composition, and interactions. However, it is limited in the quantitative analysis of complex systems, and the equipment is relatively costly. Small-angle scattering techniques can reveal detailed information about the internal structure of microemulsions, such as droplet size and morphology, but require the samples to have high purity and homogeneity. Dynamic light scattering, as a non-invasive technique, can quickly provide information on the size distribution of microemulsion droplets, although its resolution is lower for samples with broad size distributions. Cryo-electron microscopy can directly observe the structure of microemulsions at atomic resolution, although this technique faces challenges in terms of technical complexity, cost, and sample preparation. Refractive index measurement and differential scanning calorimetry can more accurately evaluate the differences between different phases of microemulsions, but they have issues with operational complexity and temperature limitations. Meanwhile, combining conductivity and zeta potential measurements can conveniently and quickly monitor the connectivity and ion strength of the water phase in microemulsions, though they cannot provide detailed information on microemulsion structure or size.

In conclusion, to fully characterize microemulsions, it is suggested that a variety of technical methods and step-by-step analysis from molecular to macroscopic scales be integrated to reveal the structure and properties of microemulsions. Through this multidimensional characterization strategy, a comprehensive understanding of microemulsion systems can be achieved, and the application and development of microemulsion science and its various industrial applications can be promoted.

### 5.2. Prospects

In the future, due to the multi-scale capabilities of these characterization techniques, a deeper integration of molecular dynamics (MD) and density functional theory (DFT) will facilitate the simulation of molecular movements and offer comprehensive temporal evolution data. This integration will also elucidate the electronic structures and intermolecular interactions within microemulsions, providing vital electronic property insights.

However, MD is constrained by time scales and system sizes, and the precision of force fields affects result credibility. DFT, on the other hand, is limited in computational scale, struggles with strong correlation effects, and has difficulty handling dynamic processes. Therefore, research should focus on developing novel methods that integrate computational modeling with artificial intelligence to streamline data interpretation and enhance predictive accuracy. Additionally, investigating new interdisciplinary applications of microemulsions, such as in nanomedicine and environmental remediation, may expand their utility and impact in various fields. Through fostering interdisciplinary collaboration and utilizing cutting-edge technologies, the future of microemulsion research holds great promise, offering exciting prospects for innovation and discovery.
